# Phylogeography of the Endangered Otago Skink, *Oligosoma otagense*: Population Structure, Hybridisation and Genetic Diversity in Captive Populations

**DOI:** 10.1371/journal.pone.0034599

**Published:** 2012-04-12

**Authors:** David G. Chapple, Alisha Birkett, Kimberly A. Miller, Charles H. Daugherty, Dianne M. Gleeson

**Affiliations:** 1 School of Biological Sciences, Monash University, Clayton, Victoria, Australia; 2 Allan Wilson Centre for Molecular Ecology and Evolution, School of Biological Sciences, Victoria University of Wellington, Wellington, New Zealand; 3 School of Biological Sciences, University of Auckland, Auckland, New Zealand; 4 Ecological Genetics Laboratory, Landcare Research, Mt. Albert, Auckland, New Zealand; Barnard College - Columbia University, United States of America

## Abstract

Climatic cooling and substantial tectonic activity since the late Miocene have had a pronounced influence on the evolutionary history of the fauna of New Zealand's South Island. However, many species have recently experienced dramatic range reductions due to habitat fragmentation and the introduction of mammalian predators and competitors. These anthropogenic impacts have been particularly severe in the tussock grasslands of the Otago region. The Otago skink (*Oligosoma otagense*), endemic to the region, is one of the most critically endangered vertebrates in New Zealand. We use mitochondrial DNA sequence data to investigate the evolutionary history of the Otago skink, examine its population genetic structure, and assess the level of genetic diversity in the individuals in the captive breeding program. Our data indicate that the Otago skink diverged from its closest relatives in the Miocene, consistent with the commencement of tectonic uplift of the Southern Alps. However, there is evidence for past introgression with the scree skink (*O. waimatense*) in the northern Otago-southern Canterbury region. The remnant populations in eastern Otago and western Otago are estimated to have diverged in the mid-Pliocene, with no haplotypes shared between these two regions. This divergence accounts for 95% of the genetic diversity in the species. Within both regions there is strong genetic structure among populations, although shared haplotypes are generally evident between adjacent localities. Although substantial genetic diversity is present in the captive population, all individuals originate from the eastern region and the majority had haplotypes that were not evident in the intensively managed populations at Macraes Flat. Our data indicate that eastern and western populations should continue to be regarded as separate management units. Knowledge of the genetic diversity of the breeding stock will act to inform the captive management of the Otago skink and contribute to a key recovery action for the species.

## Introduction

Geological and climatic processes have acted to dramatically alter the landscape of the South Island of New Zealand [1]–[3]. Throughout the Miocene the South Island was an eroded peneplain dominated by rainforest vegetation [4]–[6]. However, New Zealand straddles the boundary of the Indo-Australian and Pacific plates, and tectonic activity along the fault line in the South Island (Alpine Fault) that commenced during the Miocene, and intensified during the Pliocene, resulted in the formation of the Southern Alps (>3000 m) [2], [3], [7], [8]. As New Zealand's climate cooled during the Pliocene-Pleistocene [9], the predominant vegetation transitioned from rainforest to grassland and created an expansive subalpine and alpine zone in the mountainous regions of the South Island [1], [6], [10]. Extensive glaciers throughout the South Island were evident during glacial maxima [9], and repeated glacial cycles during the Pleistocene resulted in the continual expansion and contraction of the distribution of the resident biota [3], [11]. These processes acted to fragment the range of many species, and led to extensive speciation and phylogeographic structure within the South Island (reviewed in [3], [11]).

Anthropogenic impacts, particularly since European settlement ∼200 years ago, have resulted in the decline or extinction of many native species in the South Island [12], [13]. The introduction of a suite of mammalian predators and competitors (e.g. rodents, cats, rabbits, stoats, ferrets, weasels) has been a principal cause of many population declines, with human activities (e.g. agriculture, mining, housing developments) leading to decreased connectivity between populations due to habitat degradation and fragmentation [11], [13]–[15]. Such impacts appear to have been particularly pronounced in the tussock grassland habitats of the central Otago region of the South Island [14], [16]–[19].

**Figure 1:**
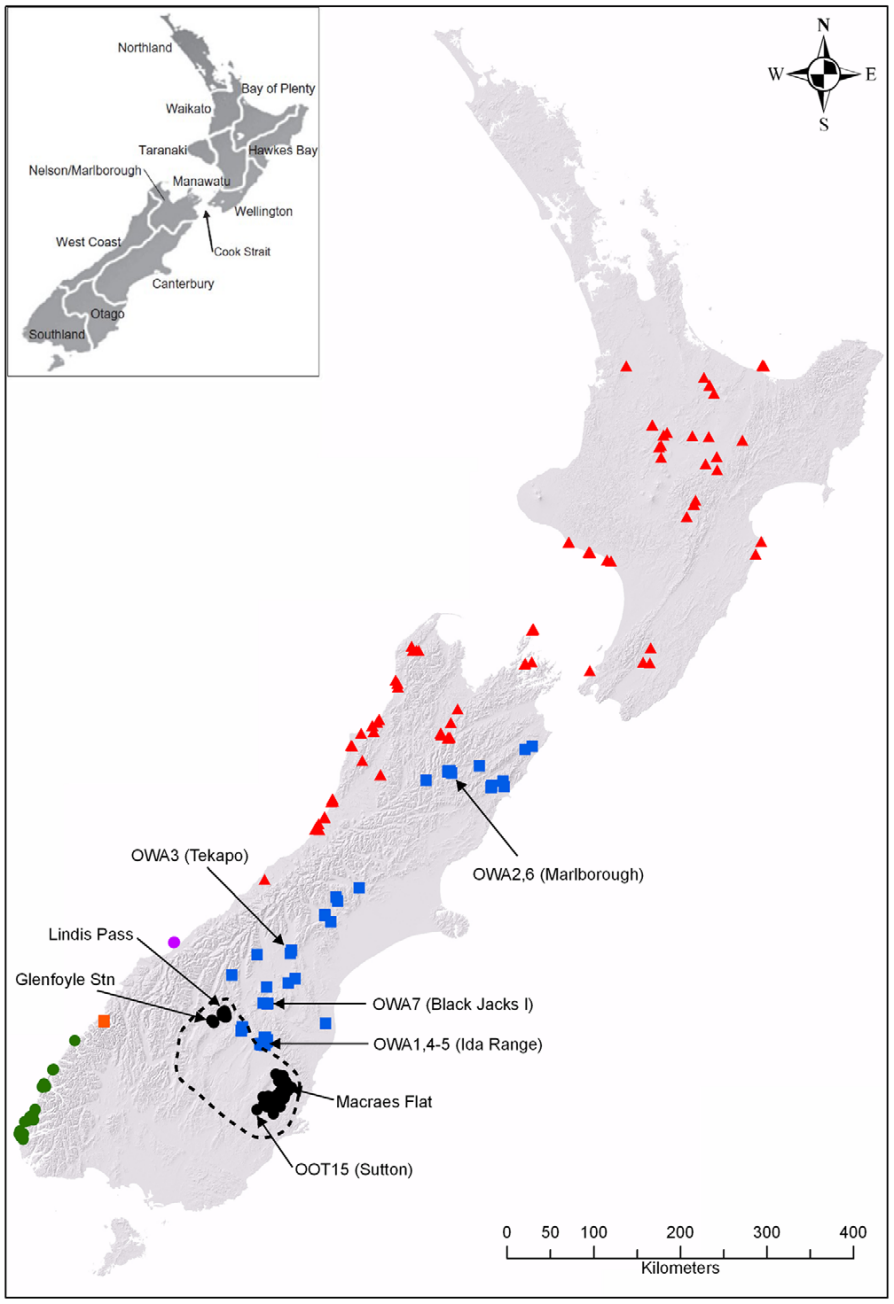
Distribution of the Otago skink and several closely related species of New Zealand skinks. The distribution of *O. otagense* (black circles), *O. waimatense* (blue squares), *O. acrinasum* (green circles), *O. infrapunctatum* (red triangles), *O. taumakae* (orange square), and *O. pikitanga* (purple circle) is indicated. Several localities mentioned in the text are highlighted, including the locations of the *O. otagense* and *O. waimatense* samples used in this study. The dashed line indicates the estimated former distribution of *O. otagense* [26]. The distribution data is adapted from the Department of Conservation's BioWeb Herpetofauna database. Inset: Major geographic regions of New Zealand.

The Otago skink (*Oligosoma otagense*), listed as Nationally Critical, is one of the most endangered vertebrates in New Zealand [20]. It is a large-sized (up to 125 mm snout-vent length: SVL) viviparous skink species that is endemic to the montane tussock grasslands of the Otago region [21]–[23]. The Otago skink is diurnal and inhabits schist rock outcrops [22], [23]. It exhibits a metapopulation structure, and habitat fragmentation has been reported to limit dispersal between rock outcrops [24], [25]. The Otago skink has experienced a substantial population decline since European settlement, and is currently estimated to inhabit only 8% of its former range [20], [22], [26], [27] (Figure 1). The species is currently restricted to two regions at the periphery of its former range: in eastern Otago between Macraes Flat and Sutton, and a few scattered populations between Lake Hawea and Lindis Pass in western Otago [22] (Figure 1). The Otago skink is still declining, with the local extinction of several populations occurring within the last 40 years [22], [26]. The largest populations occur in the Macraes Flat region, although most extant populations are extremely small (<60 individuals) [22]. It is estimated that fewer than 5000 Otago skinks remain in the wild [22], [24], [26], [28].

The conservation management of the Otago skink is focused on ∼2400 ha of land (managed by the Department of Conservation) near the Macraes Flat township [22]. A mark-recapture study has been conducted in this region since the 1990s to monitor the Otago skink populations [22]. Long-term intensive predator control (i.e. trapping, baiting, shooting) has been conducted at Macraes Flat, and several mammal-proof exclosures have been constructed at the site in an effort to increase the size of the Otago skink population [22]. Regular surveys for the Otago skink are also conducted in the western Otago populations, and a reserve was recently established to encompass the Glenfoyle Station population [22]. There are currently ∼100 Otago skinks in captivity, and several generations of offspring have been successfully bred as part of the captive breeding program [22], [23]. Although the Otago skink has been managed as a distinct taxon for several decades, it was previously considered to comprise two subspecies (*otagense*, *waimatense*; [29]) or forms (form ‘otagense’, form ‘waimatense’; [30]). *Oligosoma otagense* and *O. waimatense* (scree skink; up to 110 mm SVL) were only formally recognised as distinct species in 1997, based on body shape and colour pattern [31]. Preliminary allozyme work indicated that hybridisation may occur between the Otago skink and scree skink [32], and the two species have been observed to produce viable hybrids in captivity (D. Keall unpublished data, [33]).

Here we use mitochondrial sequence data to examine the phylogenetic history (*ND2*, *ND4*, Cytochrome b) and phylogeography (control region) of the Otago skink. In particular, we investigate the population structure within and between the two regions (eastern Otago, western Otago), determine the level of genetic diversity that is present within the captive breeding stock, and examine whether there is evidence for hybridisation between the Otago skink and scree skink.

## Materials and Methods

### Sampling

For our phylogeographic analyses, we obtained tissue samples from 63 Otago skinks, encompassing both the eastern (three sites at Macraes Flat: Falcon, Wildlife and Alistair's Gully; 45°26′S, 170°25′E) and western populations (Lindis Pass: 44°36′S, 169°40′E; Glenfoyle station: 44°43′S, 169°19′E) (Figure 1). The Wildlife and Falcon sites are within the area that is intensively managed by the Department of Conservation, while Alistair's Gully is on a privately owned farm with grazing and no predator control [23]. These samples were obtained from existing collections (National Frozen Tissue Collection [NFTC], housed at Victoria University of Wellington, New Zealand; Te Papa Tongarewa, National Museum of New Zealand, Wellington) and tissues collected by the New Zealand Department of Conservation during population surveys of the species (1998–2003). In addition, tissue samples were collected from 87 Otago skinks held in captivity (by zoos, nature parks, and private breeders) as part of the captive breeding program. As the scree skink is known to hybridise with the Otago skink in captivity [33], we also obtained tissue samples from across the range of the scree skink (7 samples from the NFTC and Te Papa Tongarewa).

A recent molecular phylogeny for the New Zealand skink fauna [34] indicates that the Otago skink is part of a monophyletic lineage that also includes *O. waimatense*, *O. acrinasum*, *O. infrapunctatum*, *O. taumakae* and *O. pikitanga* (also see [35]–[37]). Thus, samples of these five species were included, along with 15 representative *O. otagense* samples, in the broader phylogenetic analyses (Table 1). *Oligosoma polychroma* and *O. oliveri*, species from other lineages within the New Zealand skink fauna [34], were used as outgroups in the phylogenetic analyses.

**Table 1:** Locality information and GenBank accession numbers for samples used in the phylogenetic analyses.

Species	Lab Code	Museum Voucher	Tissue Code	Locality	GenBank Accession Numbers		
					*ND2*	*ND4*	*Cytb*
*Oligosoma acrinasum*	OAC1	CD826	CD826	Fiordland	EF033046	EF033060	EF071064
	OAC3	RE1839	RE1839	Resolution Island, Fiordland	EF033047	EF033061	EU567811
*O. infrapunctatum*	OIF1	CD545	CD545	Stephens Island, Cook Strait	EF033050	EF033058	EF071066
	OIF2	RE5343	FT3749	Cobden Beach, West Coast	EF033051	EF033059	EF071067
*O. otagense*	OOT1	CD1053	CD1053	Macraes Flat, Otago	EF033053	EF033064	EF071065
	OOT2	—	CD349	Macraes Flat, Otago	EF033054	EF033065	EU567814
	OOT3	—	4110	Falcon, Macraes Flat, Otago	JN999935	JN999953	JN999971
	OOT4	—	3101	Falcon, Macraes Flat, Otago	JN999936	JN999954	JN999972
	OOT5	—	0252	Falcon, Macraes Flat, Otago	JN999937	JN999955	JN999973
	OOT6	—	0324	Wildlife, Macraes Flat, Otago	JN999938	JN999956	JN999974
	OOT7	—	1210	Wildlife, Macraes Flat, Otago	JN999939	JN999957	JN999975
	OOT8	—	5505	Wildlife, Macraes Flat, Otago	JN999940	JN999958	JN999976
	OOT9	—	1201	Alistair's Gully, Macraes Flat, Otago	JN999941	JN999959	JN999977
	OOT10	—	1140	Alistair's Gully, Macraes Flat, Otago	JN999929	JN999947	JN999965
	OOT11	—	3501	Alistair's Gully, Macraes Flat, Otago	JN999930	JN999948	JN999966
	OOT12	—	OB420	Sandy Point Station, Lindis Pass, Otago	JN999931	JN999949	JN999967
	OOT13	—	OB421	Sandy Point Station, Lindis Pass, Otago	JN999932	JN999950	JN999968
	OOT14	—	OB424	Sandy Point Station, Lindis Pass, Otago	JN999933	JN999951	JN999969
	OOT15	RE5155 (S1520)	RE5155 (S1520)	2 Miles SE Sutton, Otago	JN999934	JN999952	JN999970
*O. taumakae*	OBI2	RE5237	RE5237	Taumaka Island, Open Bay Islands	EF033048	EF033062	EU567812
	OBI3	FT311	FT311	Taumaka Island, Open Bay Islands	EF033049	EF033063	EU567813
*O. waimatense*	OWA1	CD1207	CD1207	Mt Ida, Otago	EU567712	EU567742	EU567815
	OWA2	CD1209	CD1209	Wairau River, Marlborough	EF033056	EF033066	EU567816
	OWA3	—	CD760	Tekapo, Otago	JN999942	JN999960	JN999978
	OWA4	CD1208	CD1208	Mt Ida, Otago	JN999943	JN999961	JN999979
	OWA5	CD1214	CD1214	Little Mt Ida, Otago	JN999944	JN999962	JN999980
	OWA6	—	FT3011	Rag & Famish Strm Valley, Marlborough	JN999945	JN999963	JN999981
	OWA7	—	FT3012	Black Jacks Island, Lake Benmore, Otago	JN999946	JN999964	JN999982
*O. pikitanga*	SVS1	RE5315	FT7648	Sinbad Gully, Llawrenny Peaks, Fiordland	EU567713	EU567743	EU567817
*O. polychroma*	ONP1	—	FT5252	Pukerua Bay	EF033052	EF033068	EU567797
*O. oliveri*	COL1	CD1034	CD1034	Aorangi Island, Poor Knights Islands	EF033045	EF033069	EF081236

Samples with CD or FT codes were obtained from the National Frozen Tissue Collection (NFTC) housed at Victoria University of Wellington, New Zealand. Samples with RE codes were obtained from ethanol preserved specimens housed at Te Papa, National Museum of New Zealand, Wellington (S codes refer to specimens from the former Ecology Division collection, now housed at Te Papa).

### DNA extraction, amplification and sequencing

Total genomic DNA was extracted from liver, muscle, toe or tail-tip samples using a modified phenol-chloroform protocol [38] or a Bio-Rad Aqua Pure Genomic DNA Extraction Kit (Bio-Rad, Hercules CA, USA). For the phylogeographic analyses (i.e. Otago and scree skink samples) we sequenced the mitochondrial control region (∼500 bp). For the broader phylogenetic analyses, involving representative Otago skink samples and other species from the same lineage, we sequenced portions of three mitochondrial genes: *ND2* (∼600 bp), *ND4* (∼700 bp), and Cytochrome b (∼700 bp). These regions were targeted because our previous work across several taxonomic levels in New Zealand skinks has indicated useful levels of variability [34], [39]–[50]. The primers used to amplify and sequence these regions are provided in Table 2. PCR and sequencing were conducted as outlined in Berry & Gleeson [39] (control region) or Greaves et al. [40] (*ND2*, *ND4* and Cytochrome b). PCR products were purified using ExoSAP-IT (USB Corporation, Cleveland, Ohio USA). The purified product was sequenced directly using a BigDye Terminator v3.1 Cycle Sequencing Kit (Applied Biosystems) and then analysed on an ABI310 or ABI 3730XL capillary sequencer.

**Table 2:** Oligonucleotide primers used in this study.

Gene	Primer Name	Sequence (5′-3′)	5′ Position	Source
***ND2***	L4437	AAGCTTTCGGGCCCATACC	3833	[92]
	ND2r102	CAGCCTAGGTGGGCGATTG	4432	[93]
***ND4***	ND4I	TGACTACCAAAAGCTCATGTAGAAGC	10771	[94]
	ND4R-NZ	CCAAGRGTTTTGGTGCCTAAGACC	11670	[40]
	tRNA-Leu	TACTTTTACTTGGATTTGCACCA	11691	[94]
**Cytochrome b**	mtD-25	CCATCCAACATCTCAGCATGATGAAA	14202	[95]
	SkCytBR	TAGGCAAANARRAAGTAYCAYTCTGG	14940	[40]
**Control region**	tRNAp-L	GCTAACCCCTCGTCACTAACTCC		[39]
	CR-Rev-3	GCACCTGACACTAGTAACGG		This study

The letters L and H refer to the light and heavy strands. Values in ‘5’ position' refer to the position of the 5′ position in the complete *Eumeces egregius* mtDNA sequence [96].

Sequence data were edited using ContigExpress in Vector NTI Advance v9.1.0 (Invitrogen), and aligned using the default parameters of Clustal X v1.83 [51]. We translated all coding region sequences to confirm that none contained premature stop codons. Sequence data were submitted to GenBank [GenBank: JN999929-JN999994] (Table 1).

### Phylogenetic analyses

The concatenated *ND2*, *ND4* and cytochrome b sequence data was used for the phylogenetic analyses. Maximum Likelihood (ML) and Bayesian tree building methods were used. We used Modeltest 3.7 [52] to identify the most appropriate model of sequence evolution based on the Akaike information criterion (AIC) [53]. Modeltest, conducted in PAUP* 4.0b10 [54], was also used to estimate base frequencies, substitution rates, the proportion of invariable sites (I) and the among-site substitution rate variation (G) [55]. These values were then used as settings in PhyML 3.0 [56] to generate a ML tree with 500 bootstraps.

MrBayes 3.1.2 [57] was used to complete Bayesian analyses. We used Modeltest to determine the most appropriate model of sequence evolution for our dataset. We ran the Bayesian analysis for five million generations, sampling every 100 generations (i.e. 50,000 sampled trees). We ran the analysis twice, using four heated chains per run. We discarded the first 25% of samples as burn-in and the last 37,500 trees were used to estimate the Bayesian posterior probabilities. The program Tracer 1.5 [58] was used to check for chain convergence and mixing.

Bootstrap values (500 ML bootstraps) and Bayesian posterior probabilities were used to assess branch support. We considered branches supported by bootstrap values of 70% or greater [59], and/or posterior probability values greater than or equal to 0.95 [60] to be supported by our data.

We estimated the divergence time of the eastern and western Otago skink populations using an evolutionary rate of 1.3–1.63% sequence divergence per million years, based on mitochondrial DNA calibrations from other squamate reptile groups (1.3%, [61]; 1.42–1.63%, [62]; 1.55%, [63]; 1.62%, [64]; 1.63%, [65]). A strict molecular clock (0.0065–0.00815 substitutions per site per million years), implemented in BEAST v1.6.1 [66], was used to estimate the divergence times. The New Zealand skink lineage is estimated to have originated ∼20 mya [34], [67], and this information was used as the maximum age of the tree root. A GTR+I+G model of evolution was employed with a speciation (Yule) tree prior. The analysis was run twice, with 20 million generations per run (total 40 million generations after the two runs were combined using LogCombiner v1.6.1). The output was viewed in Tracer to check that stationarity had been reached, and ensure that the effective sample size (ESS) exceeded 200 [66].

### Phylogeographic analyses: molecular diversity and population structure

The control region dataset was used for the phylogeographic analyses. Estimates of genetic diversity within Otago skink populations (number of haplotypes, *h*; haplotypic diversity, *Hd*; number of polymorphic sites, *S*; nucleotide diversity, π) were calculated in DnaSP v4.50 [68]. Tamura-Nei (TrN)-corrected genetic distances within and among populations were calculated in MEGA 4 [69]. Haplotype networks were created using TCS v1.21 [70]. Genetic differentiation among populations was estimated in Arlequin v3.5 [71]. Pairwise Φ_ST_ values (an analogue of Wright's fixation index *F*_ST_) were calculated to estimate among population differentiation. We conducted hierarchical Analysis of Molecular Variance (AMOVA; [72]) to investigate the partitioning of genetic variation within and among populations and regions. Both tests used TrN genetic distances. Significance levels of all the estimated values were calculated by 10,000 permutations, and adjusted according to the Bonferroni correction procedure [73] for multiple pairwise comparisons as described by Holm [74].

We used Tajima's *D* [75], Fu's *F*s statistic [76] (calculated in Arlequin) and mismatch distributions to test for signatures of population expansion (significance levels were calculated by 10,000 permutations). Significant and negative Tajima's *D* and Fu's *F*s statistic values are indicative of possible population expansion. Mismatch frequency histograms were plotted in DnaSP to determine whether the populations exhibited evidence of spatial range expansion or a stationary population history [75]. A smooth bell shape signifies either population expansion or spatial range expansion, whereas a multimodal distribution represents a long history *in situ* [77]–[80]. To distinguish between these two types of distribution, a raggedness index (RI, sum of the squared difference between neighbouring peaks) and the sum of squared deviations (SSD) between the observed and expected mismatch were calculated using the methods of Schneider & Excoffier [81] in Arlequin. The spatial expansion hypothesis (both RI and SSD) was tested using a parametric bootstrap approach (200 replicates).

## Results

### Phylogeny and divergence time estimates

The edited alignment comprised 1834 characters (550 bp *ND2*, 671 bp *ND4*, 613 bp Cytochrome b), of which 588 (32%) were variable and 438 (24%) were parsimony-informative. For the ingroup only, the alignment contained 487 (27%) variable characters, of which 397 (22%) were parsimony-informative. Base frequencies were unequal (A = 0.3026, T = 0.2608, C = 0.3016, G = 0.1350), but a χ^2^ test confirmed the homogeneity of base frequencies among sequences (df = 90, *P* = 1.0).

**Figure 2:**
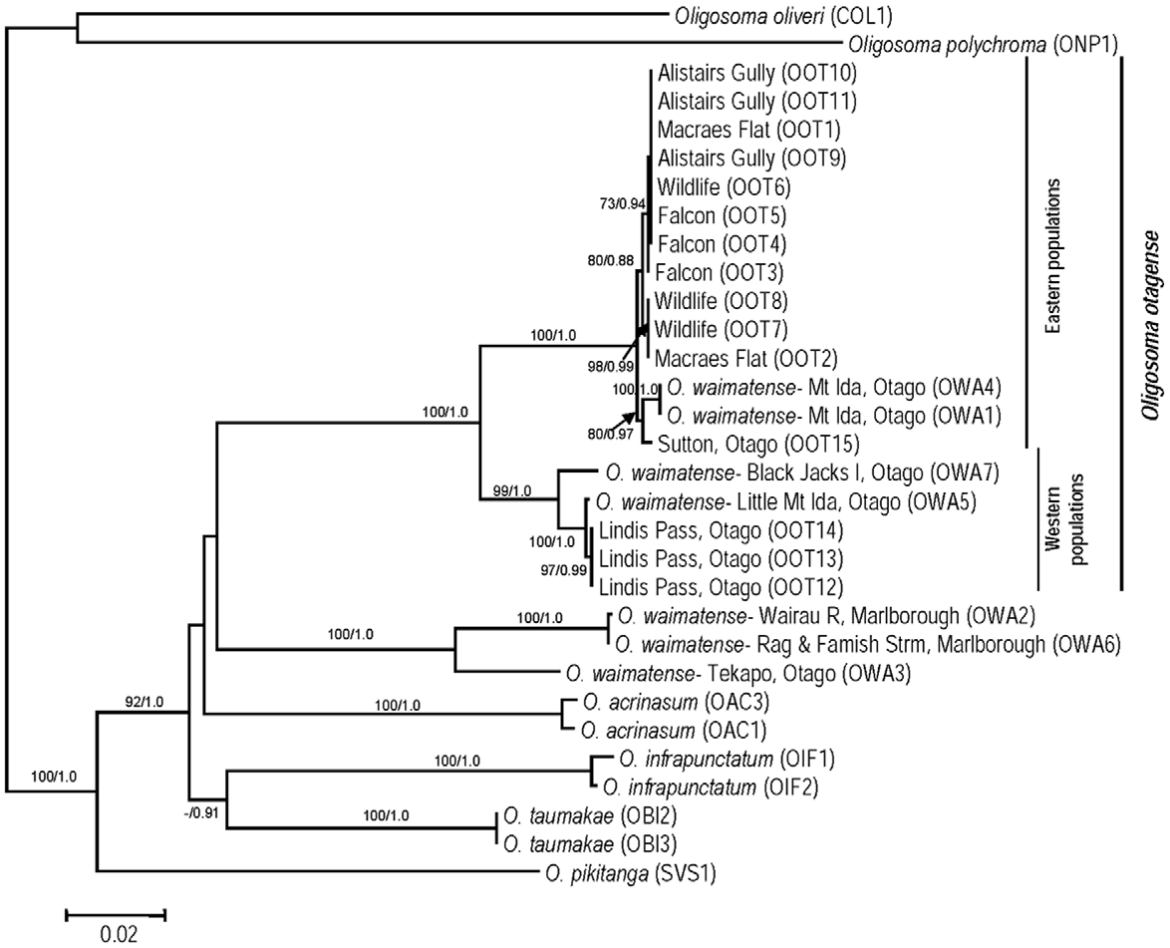
Maximum Likelihood (ML) phylogram for the New Zealand skink lineage containing the Otago skink (*Oligosoma otagense*). The lineage contains five other species: *O. waimatense*, *O. acrinasum*, *O. infrapunctatum*, *O. pikitanga*, and *O. taumakae*. The phylogeny is based upon 1834 bp of mitochondrial DNA sequence data (*ND2*, *ND4* & Cytochrome b). Two measures of branch support are indicated with ML bootstraps (500 replicates) on the left and Bayesian posterior probabilities on the right (only values over 50 and 0.7, respectively, are shown).

The AIC from Modeltest supported the GTR+I+G substitution model as the most appropriate for our dataset. Parameters estimated under this model were: relative substitution rates (A↔C = 2.16, A↔G = 38.47, A↔T = 1.50, C↔G = 1.11, C↔T = 19.04, relative to G↔T = 1.00), proportion of invariable sites (0.6022), and gamma distribution shape parameter (4.132). The topologies of the ML and Bayesian trees were identical, therefore we present the optimal ML tree (−ln *L* = 7317.86919) with ML bootstrap (BS) values and Bayesian posterior probabilities (PP) indicating branch support (Figure 2).

There is extremely strong support for the monophyly of *O. acrinasum*, *O. infrapunctatum*, *O. pikitanga*, and *O. taumakae* (100 BS and 1.0 PP in all cases; Figure 2). Although two distinct genetic lineages were evident within *O. otagense*, representing eastern (100 BS, 1.0 PP) and western populations (99 BS, 1.0 PP), several *O. waimatense* individuals from the southern limit of its distribution grouped with the eastern (Mt Ida population; OWA1, 4) or western (Little Mt Ida and Black Jacks Island populations; OWA5, 7) lineages of *O. otagense* (Figure 2). In contrast, the *O. waimatense* individuals from the remainder of the distribution (OWA2, 3, 6) formed a well-supported lineage (100 BS, 1.0 PP), which was genetically divergent (11.5% sequence divergence) from both *O. otagense* lineages (Table 3). The mean genetic divergence among recognised species (excluding OWA1, 4–5, 7) was 9.4–13.4% (Table 3). The divergence between the eastern and western populations of *O. otagense* was 4.9% and estimated to have occurred 3.7 mya (95% highest posterior density [HPD] confidence interval: 2.8–4.6 mya).

**Table 3:** Mean model-corrected genetic distances (*ND2*, *ND4*, Cytochrome b) between the Otago skink and several closely-related species.

	*O. otagense*- E	*O. otagense*- W	*O. waimatense*	*O. acrinasum*	*O. infrapunctatum*	*O. pikitanga*	*O. taumakae*
*O. otagense*- E	—						
*O. otagense*- W	0.049	—					
*O. waimatense*	0.115	0.115	—				
*O. acrinasum*	0.117	0.110	0.106	—			
*O. infrapunctatum*	0.115	0.111	0.119	0.116	—		
*O. pikitanga*	0.134	0.127	0.125	0.130	0.132	—	
*O. taumakae*	0.106	0.097	0.110	0.094	0.100	0.114	—

### Phylogeographic analyses: molecular diversity and population structure

**Figure 3:**
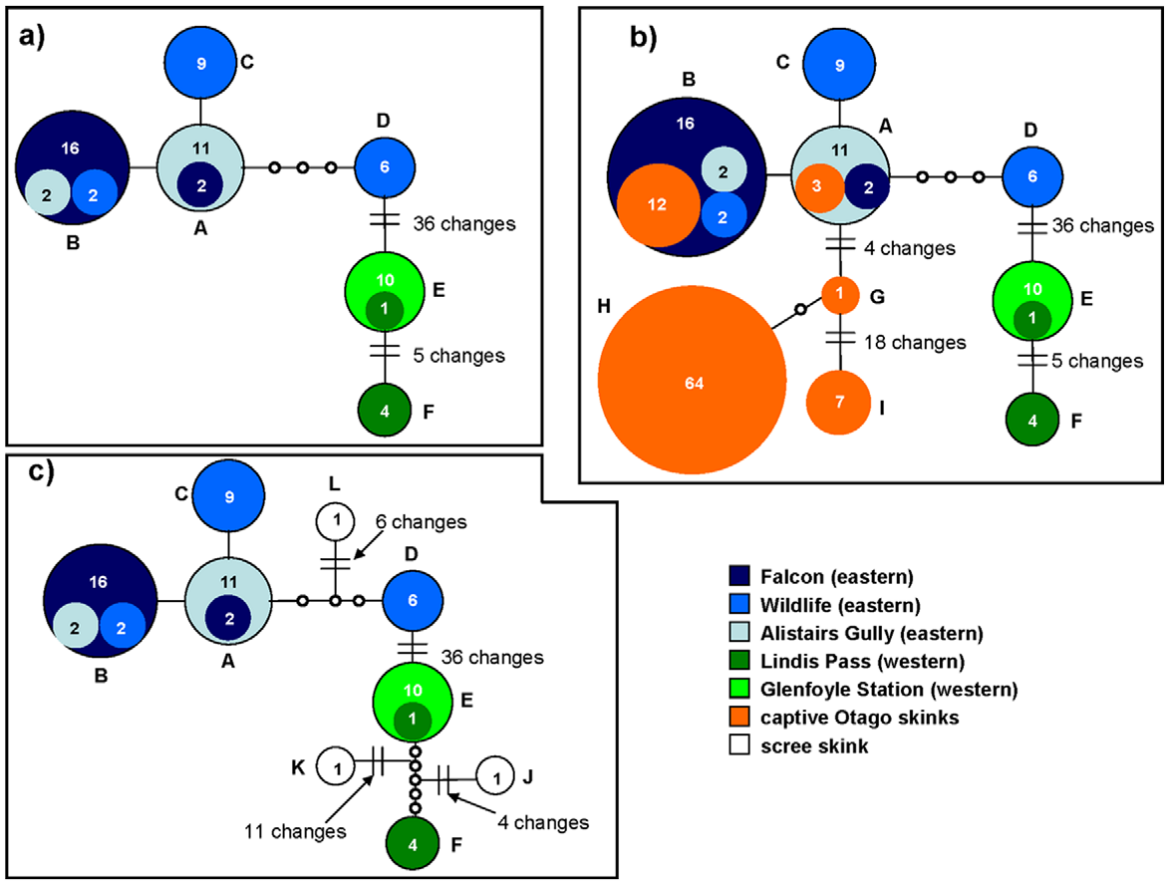
*Control region* haplotype network for the Otago skink. Each circle represents one haplotype and the size indicates the number of individuals with each haplotype. The lines indicate one base pair change between sequences. The different colours indicate the population(s) in which each haplotype is found. A) Wild Otago skink populations, B) Wild and captive Otago skinks, C) Wild Otago skinks and introgressed scree skinks.

The edited control region alignment comprised 419 characters, with 46 variable sites within the six haplotypes identified within the Otago skink (Table 4, Figure 3a). No haplotypes were shared between the eastern and western populations (Figure 3a). Four haplotypes were found to occur within the Macraes Flat region, with one present in all three populations (Haplotype B), another present at Falcon and Alistair's Gully (Haplotype A), and a further two (Haplotypes C and D) found exclusively at the Wildlife site (Table 4, Figure 3a). Two haplotypes were identified in the western region (Haplotypes E and F), with both occurring in the Lindis Pass population, but only Haplotype E was present in the Glenfoyle Station population (Table 4, Figure 3a). Consistent length variation was observed between the control region haplotypes in the eastern (419 bp) and western (415 bp) regions of the Otago skinks range. Both haplotype and nucleotide diversity was generally low within each population (Table 4).

**Table 4:** Estimates of genetic diversity (mitochondrial *control region*) within Otago skink populations and regions.

Pop/region	*n*	*h*	*Hd*	*M*(*S*)	π	Tajima's *D*	Fu's *F*s	RI	SSD
**Eastern**	48	4 (A–D)	0.717	6(6)	0.004	0.663	2.539	0.094	0.029
Falcon	18	2 (A,B)	0.209	1(1)	0.001	−0.529	−0.011	0.382	0.001
Wildlife	17	3 (B–D)	0.618	6(6)	0.006	1.732	4.005	0.653	0.165*
Alistair's Gully	13	2 (A,B)	0.282	1(1)	0.001	−0.274	0.240	0.270	0.001
**Western**	15	2 (E,F)	0.419	6(6)	0.006	1.269	5.710	0.689	0.130
Lindis Pass	5	2 (E,F)	0.400	6(6)	0.006	−1.146	3.022	0.680	0.118
Glenfoyle Stn	10	1 (E)	NA	NA	NA	NA	NA	NA	NA
**Overall**	63	6 (A–F)	0.805	47(46)	0.039	NA	NA	NA	NA
**Captive animals**	87	5 (A,B,G–I)	0.437	23(22)	0.011	NA	NA	NA	NA

*n* = sample size, *h* = number of haplotypes (the specific haplotypes present are indicated), *Hd* = haplotypic diversity, *M* = total number of mutations, *S* = number of segregating (polymorphic) sites, π = nucleotide diversity, RI = raggedness index, SSD = sum of squared deviations. Asterisks indicate significant Tajima's *D*, Fu's *F*s statistic, RI and SSD values.

Substantial genetic differentiation was evident between the two regions (10.6–11.5% sequence divergence, Table 5; pairwise Φ_ST_ = 0.958), with 36 variable sites separating the eastern and western populations (Figure 3a). Although the level of genetic differentiation among populations within each region was lower (0.2–1.5% sequence divergence, Table 5), significant pairwise Φ_ST_ values were found among all five populations (Table 6). The AMOVA confirmed that most genetic variation was partitioned among regions (94.60%), rather than among populations in each region (3.09%), or within populations (2.31%). There was no strong evidence for recent range expansion in any of the five populations (Table 4).

**Table 5:** Mean TrN genetic distances between *control region* haplotypes.

	Hap A	Hap B	Hap C	Hap D	Hap E	Hap F	Hap G	Hap H	Hap I	Hap J	Hap K	Hap L
Hap A	—											
Hap B	0.002	—										
Hap C	0.002	0.005	—									
Hap D	0.010	0.012	0.012	—								
Hap E	0.109	0.106	0.106	0.106	—							
Hap F	0.115	0.112	0.112	0.112	0.015	—						
Hap G	0.012	0.014	0.014	0.017	0.115	0.121	—					
Hap H	0.017	0.019	0.019	0.017	0.109	0.115	0.005	—				
Hap I	0.050	0.047	0.053	0.053	0.122	0.127	0.045	0.045	—			
Hap J	0.121	0.118	0.118	0.118	0.015	0.015	0.127	0.121	0.127	—		
Hap K	0.115	0.112	0.112	0.112	0.030	0.035	0.120	0.115	0.131	0.040	—	
Hap L	0.017	0.020	0.020	0.017	0.106	0.112	0.012	0.007	0.050	0.118	0.112	—

**Table 6:** Pairwise Φ_ST_ among the five Otago skink populations.

	Falcon	Wildlife	Alistair's Gully	Lindis Pass	Glenfoyle Station
Falcon	—				
Wildlife	0.477*	—			
Alistair's Gully	0.687*	0.284*	—		
Lindis Pass	0.986*	0.942*	0.983*	—	
Glenfoyle Station	0.996*	0.961*	0.996*	0.835*	—

Asterisks indicate statistical significance following Bonferroni correction (adjusted significance level 0.005).

Five haplotypes, each originating from the eastern region, were identified from the 87 captive Otago skinks (Tables 1 and 5; Figure 3b). Two of these corresponded to haplotypes currently present at Macraes Flat (Haplotypes A, B), but the other three haplotypes (Haplotypes G, H and I) do not appear to occur in the populations that are intensively managed by the Department of Conservation (Figure 3b). Indeed, the majority (83%) of captive Otago skinks were found to have haplotypes (G: n = 1; H: n = 64; I: n = 7) that are not evident in the managed sites at Macraes Flat (Figure 3b). The level of genetic differentiation among these three haplotypes from captive animals and those present at Macraes Flat was low (G: 1.2–1.7%; H: 1.7–1.9%) to moderate (I: 4.7–5.3%) (Table 5).

Three unique control region haplotypes were identified in the four scree skinks (Haplotype J: OWA5; Haplotype K: OWA7; Haplotype L: OWA1, OWA4) which appeared, based on our phylogenetic analyses, to derive from lineages that had experienced past introgression with the Otago skink (Table 1; Figures 2 and 3c). This introgression appears to have occurred with both the eastern (Haplotype L) and western (Haplotypes J and K) Otago skink populations (Figure 3c). None of the scree skink haplotypes are currently found in the wild or captive Otago skink populations (Figure 3), with low to moderate levels of genetic differentiation to the Otago skink haplotypes present in the respective regions (Table 5).

## Discussion

Our study indicates that the Otago skink exhibits substantial genetic divergence (9.7–13.4%) from its closest relatives in the South Island (*O. acrinasum*, *O. infrapunctatum*, *O. pikitanga*, *O. taumakae*, *O. waimatense*). The diversification of this lineage has previously been estimated to have occurred during the late Miocene [34], corresponding to the commencement of tectonic activity along the Alpine Fault and the uplift of the Southern Alps [2], [3], [7], [8]. At present, these six species have non-overlapping distributions in the South Island (Figure 1), which may indicate that the diversification of this lineage during the late Miocene occurred through allopatric speciation [34].

However, both our phylogenetic and phylogeographic datasets indicate that hybridisation has occurred historically (i.e. introgression) between the Otago skink and scree skink in the northern Otago-southern Canterbury region. Incomplete lineage sorting is unlikely to explain our results given the deep divergence between the two species, the restriction of the pattern to the southern end of the scree skinks range, and the ability for the two species to produce viable hybrids in captivity. Hardy [30] hypothesized that the divergence between these two species occurred during the Pleistocene, with the two species retreating to isolated refugia in Otago (Otago skink) and Canterbury (scree skink) during glacial maxima, and expanding their ranges during interglacials. Although the divergence between the two species occurred earlier in the late-Miocene [34], they appear to have hybridised when they came into secondary contact in the northern Otago-southern Canterbury region during Pleistocene interglacials. Our data suggests that the scree skink hybridised with both the eastern and western lineages of the Otago skink. This might have involved Otago skink populations that have since gone locally extinct (i.e. scree skinks were found to have haplotypes that are not currently present in the wild or captive Otago skink populations). Alternatively, the scree skink haplotypes may have diverged since the introgression event. Interestingly, we detected no evidence of scree skink haplotypes occurring in either the eastern or western lineages of the Otago skink. Although the two species do not currently occur in sympatry, they have the capacity to produce viable hybrids in captivity (D. Keall unpublished data, [33]). This not only has important implications for selection of breeding stock for the Otago skink captive breeding program, but also for the conservation management of both species in the wild (e.g. [82]). Future studies should use nuclear markers (e.g. nuclear genes, microsatellites) to further investigate the patterns of introgression between the Otago and scree skink.

Two main lineages were evident within the Otago skink, with the divergence between the populations in eastern Otago and western Otago occurring during the Pliocene (mean 3.7 mya, range 2.8–4.6 mya). Phylogeographic breaks of an equivalent age have been reported in this region for the sympatric grand skink (*O. grande*, 3.8 mya; [39]), McCann's skink (*O. maccanni*, 3.6 mya; [45]), green skink (*O. chloronoton*, 5.3 mya; [40]), and cryptic skink (*O. inconspicuum*, 2.7 mya; [50]). These divergences in South Island skink species are concordant with the Nevis-Cardrona fault system (marked by the Cardrona and Nevis rivers) that has been active since the Miocene and delineates a topographic boundary between eastern Otago (undulating grassland habitats) and western Otago (deeply-eroded rugged mountainous habitat) (reviewed in [83]). However, the extinction of the Otago skink from this boundary region makes it difficult to identify the exact position of this split. The consistent length variation in the control region haplotypes, and the lack of shared haplotypes between eastern and western Otago, indicates that there has been no geneflow between the remnant Otago skink populations in the two regions for a substantial period of time. This is exemplified by our finding that the divergence between the eastern and western regions accounts for ∼95% of the genetic variation within the Otago skink. However, given the recent extinction of the populations in central Otago it is unknown what pattern of geneflow (e.g. isolation by distance) was evident throughout the species range prior to its decline.

Substantial genetic structuring was also evident within both the eastern (three Macraes Flat populations) and western (Lindis Pass, Glenfoyle station) populations of the Otago skink. Despite this, the presence of shared haplotypes within each region indicates that there has been recent geneflow among adjacent populations. This pattern of population structuring is consistent with that observed in the grand skink [39], a species that has similar habitat preferences (i.e. rocky outcrops in montane tussock grassland), and a near identical distribution (both historical and current distribution) and conservation status (Nationally Critical) [20], [22], [84]. Similar to the recommendations for the grand skink [39], we suggest that the eastern and western populations of the Otago skink should continue to be treated as separate management units (e.g. [85]–[87]). Although a reserve was recently established at Glenfoyle Station and Otago skink survey work is regularly conducted in the western populations, the populations in the Macraes Flat region are subject to the most intensive management by the Department of Conservation [22], [84]. Our study indicates that these managed populations (Wildlife, Falcon) contain all of the haplotypes known from the eastern region, but only a small proportion of the species total genetic diversity.

Considerable genetic and haplotypic diversity was evident in the Otago skinks in the captive breeding program. All captive animals had haplotypes that originated from the eastern region. The initial founders of the captive breeding program were sourced from near Sutton in the 1980s–1990s (A. Hutcheon, personal communication), and this accounts for most captive animals having haplotypes (i.e. Haplotypes G, H, I) that originate from outside of the intensively managed populations at Macraes Flat. Given the recent population declines of the Otago skink in the Sutton region [22], [26], the captive breeding stock may contain haplotypes that have since gone extinct in the wild. Although the recent additions to the captive breeding program have been sourced from the Macraes Flat region, only two (A, B) of the four haplotypes from the region are represented in the captive populations.

The Department of Conservation is in the process of establishing a separate captive breeding program for the western populations of the Otago skink (A. Hutcheon, personal communication), and continues to implement in-situ conservation management actions at the Glenfoyle station reserve [22]. As these populations represent a distinct management unit, the captive breeding stock should help to safeguard the full extent of genetic diversity that is evident in the species. Indeed, the current recovery plan aims to maximise the genetic diversity present in captivity populations to enable the reintroduction of individuals to the wild [22]. However, as discussed previously by Connolly & Cree [23], the Otago skink breeding program should consider the phenotype, or ‘quality’ (i.e. morphology, parasite load, body condition, growth rate, sprint speed), of the captive animals, rather than just the genetic diversity represented in the breeding stock.

### Conclusions

The Otago skink has experienced a drastic reduction in its distribution over the last ∼200 years and now persists in two remnant regions in the periphery of its original distribution [22], [26]. A consistent pattern in conservation biology has been that during range decline, most species are able to persist in the peripheral regions of their historical range after the local extinction of the populations from the interior of the geographic distribution [88], [89]. Compared to the core of the species range, populations occurring at range margins are usually exposed to suboptimal environmental conditions (reviewed in [90]), and therefore remnant populations may not be representative of the species habitat preferences and population genetic structure (reviewed in [89]). This may be true in the Otago skink, as the haplotypes present in the captive animals and the introgressed scree skinks indicate that there has been a significant recent reduction in genetic diversity present across the species range. It may also indicate that the Otago skink currently persists in suboptimal or atypical habitats, and might benefit from translocations (within predator-proof exclosures) to areas near the core of the species' historic range. Hybridisation with the scree skink is another potential concern for the future conservation management of the Otago skink. Although there is currently limited opportunity for hybridisation between the two species as their ranges do not overlap, as many species are anticipated to shift their distributions as a result of climate change [91], there is the potential for hybridisation in the future if the scree skink expands its range southwards into the distribution of the Otago skink.
